# Increased minimum alveolar concentration-awake of Sevoflurane in women of breast surgery with sleep disorders

**DOI:** 10.1186/s12871-020-0931-3

**Published:** 2020-01-20

**Authors:** Yuanyuan Cao, Lei Zhang, Xiaohui Peng, Yun Wu, Qunlin Zhang, Erwei Gu, Ye Zhang

**Affiliations:** 1 Department of Anesthesiology, Second Affiliated Hospital of Anhui Medical University, 678 Furong Rd, Hefei, Anhui China; 2 Department of Anesthesiology, First Affiliated Hospital of Anhui Medical University, 218 Jixi Rd, Hefei, Anhui China; 3 Department of Pharmaceutical Analysis, Anhui Medical University, Hefei, Anhui China

**Keywords:** Sevoflurane, MACawake (minimum alveolar concentration of awake), Orexin-A, Sleep disorders

## Abstract

**Background:**

Sleep disorders are commonly encountered in clinic. Evidences showed that sleep deprivation may modulate the effectiveness of general anesthetics in rats. However, this phenomenon has not been explored in humans. The study aimed to investigate whether the hypnotic potency of sevoflurane in patients with sleep disorders differ from patients with normal sleep habits.

**Methods:**

We recruited 44 patients scheduled for elective breast surgery and eventually analyzed 38 patients, including 19 subjects with normal sleep habits and 19 subjects with sleep disorders. According to the Dixon ‘up-and-down’ design, patients received sevoflurane at preselected concentrations starting at 1.0 vol%. After a steady-state period, a verbal command for testing awakening was performed. Based on the negative or positive response to the verbal command, we decreased or increased the concentration of sevoflurane by 0.2 vol% in the next patient accordingly. Plasma orexin-A was also measured before observation.

**Results:**

The MACawake of sevoflurane was 0.80% [95% confidence interval (CI), 0.683–0.926%] in the sleep disordered group vs 0.60% [95% CI, 0.493–0.689%] in the control group. The relative median potency between groups was 0.750 (95% CI, 0.236–0.969). Patients with sleep disorders had significantly higher orexin-A levels than control (72.17 ± 18.24 vs. 36.16 ± 14.18 pg/mL). A significant, positive relationship was detected between orexin-A level and probability of awakening (OR = 1.081, 95% CI is 1.020–1.146, *P* = 0.008).

**Conclusions:**

MACawake of sevoflurane is higher in mild-aged women of breast surgery with sleep disorders compared to those with normal sleep habits. The increased anesthetic requirement may be related to changes of orexin-A levels. These findings suggest that sleep may have a potential impact on clinical anesthesia, including changes of sensitivity to anesthetics or postoperative complications. Further research is needed to confirm this hypothesis.

**Clinical trial registration:**

Chinese Clinical Trial Registry (ChiCTR1800016022), date of registration 07 May 2018.

## Background

Sleep disorders such as insomnia are commonly encountered in clinic because of fear, anxiety, pain, or a disruptive environment. Patients with sleep disorders are more likely to experience postoperative complication like delirium [1, 2]. It has been shown that sleep deprivation can modulate the effectiveness of general anesthetics in rats, including hypersensitivity to induction of anesthesia, or delayed emergence from anesthesia [ 3, 4]. However, the mechanism of sleep changes affect general anesthesia remains unclear.

It has been speculated that common neural pathways between natural sleep and general anesthesia may regulate emergence from anesthesia [5]. Orexins, especially orexin-A, play a crucial role in the promotion and maintenance of wakefulness during anesthesia emergence [6, 7]. One previous study showed that a reduction of Orexin-A is responsible for prolonged emergence from isoflurane anesthesia in rats subjected to sleep deprivation [4]. However, this theory has yet to be confirmed in humans. Therefore, we hypothesized that sleep disorders along with a change of orexin-A might modulate the hypnotic potency of anesthetics in humans.

To test this, we assessed the minimum alveolar concentration (MAC) of inhaled anesthetic agents to achieve a 50% probability of nonresponsive to a stimulus (MACawake), which provides a measure of hypnotic potency. Considering that sevoflurane is the most commonly used volatile anesthetics for general anesthesia and sedation, the aim of our study was to determine whether the MACawake of sevoflurane in women with sleep disorders are different to those with normal sleep patterns (control). Also, we aimed to determine a correlation between the probability of awaking and plasma concentrations of orexin-A.

## Methods

### Participants

This study was approved by our local Institutional Ethical Committee (First Affiliated Hospital of Anhui Medical University, and informed consent was obtained preoperatively from each patient. We enrolled 44 women aged 40–60 yrs. undergoing elective breast surgery using general anesthesia from May 2018 to September 2018 at First Affiliated Hospital of Anhui Medical University, Hefei, China.

All participants were classified by the American Society of Anesthesiologists physical status classification of I or II, without dysaudia. Patients were excluded if they presented with any of these criteria: anemia, hypoxia, hypotension, hypercapnia, abnormal acid–base status, electrolyte disturbances, fever, or obesity (body mass index > 30 kg m^2^); associated with any neurological disease and psychotropic medications; daily alcohol consumption; history of drug dependence or use of over-the-counter sleep medications within the previous 6 months. All patients gave written, informed consent.

The following inclusion criteria were used for subjects with sleep disorders [8]: difficulty initiating or maintaining sleep≥6 months, with Pittsburgh Sleep Quality Index (PSQI) ≥7, according to the Diagnostic and Statistical Manual of Mental Disorders (DSM-V) criteria [9]; Hamilton Anxiety Scale (HAMA) < 7; and Hamilton Depression Scale (HAMD) < 7. The following criteria were used for normal sleepers [8]: no history or evidence of sleep disorders; PSQI < 7, HAMA and HAMD scores less than 7.

All subjects were interviewed and assessed for PSQI, HAMA, and HAMD by an trained anesthesiologist on the day before surgery. Written informed consent was obtained at the same time.

### Up and down method

All patients had fasted for 8 h and received no sedative or anesthesia drugs prior to the observation. Standard monitoring was conducted throughout the observation, including electrocardiogram, noninvasive blood pressure, heart rate, pulse oximeter, and inhaled gas analysis (carbon dioxide and sevoflurane).

General anesthesia was induced by breathing 8% sevoflurane (Maruishi Pharmaceutical Co, Ltd., Osaka, Japan) mixed with 100% oxygen at a flow rate of 6 l min^− 1^ through a semi-closed face mask, delivered through a semi closed circuit system (Sevotec 7; Datex- Ohmeda Inc., Madison, WI, USA). The inspired and end tidal concentrations of sevoflurane and end tidal carbon dioxide in mmHg were sampled through a tube connected to the distal end of the mask and analyzed by a gas analyzer (Philips G60 Series Patient Monitor, Philips, Böblingen, Germany).

After loss of the lash reflex, the target concentration of the first patient was adjusted to 1.0% in 100% oxygen at a flow rate of 2 L min^− 1^ in both two groups. After maintaining for 15 min, verbal commands were given to test awakening by an independent observer who was blinded to the design and the aim of the study. The observer asked the patient to open their eyes with a normal tone, and repeated 3 times. The response of each patient was record as ‘no response’ or ‘response’. According to the “up and down” method [10], the target concentration of next patient was dependent on the response of the previously tested patient. If the previous patient responded, the target concentration was set at 0.2% higher, but the target concentration was set at 0.2% lower if the previous patient did not respond to the command.

The operating room was kept quiet and at a temperature ranging from 24 to 26 °C. The warmed lactated Ringer’s was injected at a rate of 8–10 mL kg^− 1^ h^− 1^. Ventilation was assisted if the end-tidal carbon dioxide (ETCO_2_) level was > 45 mmHg, or if the tidal volume was too low (< 500 mL). Phenylephrine 40 μg was administered i.v. if necessary to maintain MAP. A previous observer blinded to the study recorded the data. After the observation, anesthesia was deepened appropriately for tracheal intubation and surgery.

### Measurement of orexin-a

Blood samples (5 mL) were collected into EDTA tubes before observation for blood gas analysis and then transferred into centrifuge tubes containing aprotinin. The blood samples were centrifuged at 1600 rpm for 15 min at 4 °C to separate plasma and then stored at − 80 °C for assay later. Plasma orexin-A was measured by a Chemiluminescent EIA kit (Phoenix pharmaceuticals Inc., Burlingame, California, USA).

### Statistical analysis

All statistical analyses were performed using SPSS software version 19.0 (SPSS for MAC, Chicago, IL, USA). Patient characteristic data were presented as means (SD). Patient characteristics, blood glucose, liquid volume and ETCO_2_ were compared between groups by independent-samples t-test or nonparametric test.

Up and down sequences were analyzed by the probit test, which enabled estimation of MACawake with 95% CI of the mean. The calculated MACawake of sevoflurane between groups was compared using the estimate of relative median potency, which is the ratio of sevoflurane concentration needed to obtain a 50% probability of being awake in each group. Logistic regression was conducted to test the correlation of probability of awakening and Orexin-A. *P* values of < 0.05 were considered statistically significant.

In our previous study, we determined a MACawake of 0.67% for patients 50 yrs. old [11]. We conducted an a priori power analysis to detect a difference of 0.2% (SD = 0.2) in the concentration of sevoflurane. On the assumption of power of 0.80 and a type I error rate of 0.05, we needed to enroll 16 patients for each group.

## Results

**Fig. 1:**
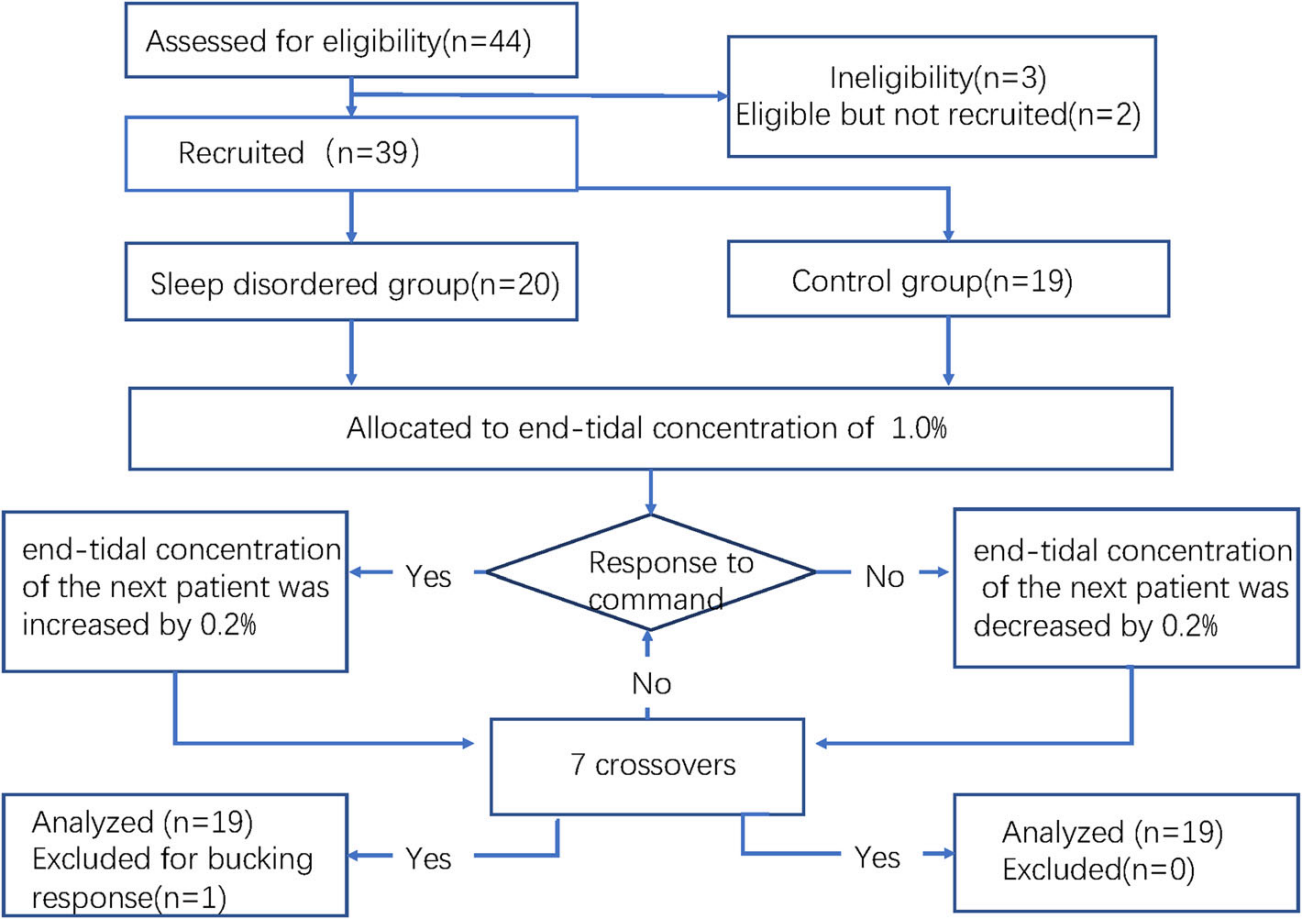
Flow diagram for the Dixon up and down method. Five patients were excluded from observation (2 without consent and 1 with obesity and 1 with severe anemia and 1 for midazolam was used before observation); one patient was excluded from analysis for serious cough

Forty-four patients completed the investigation as shown in Fig. 1. Six patients were excluded from the study. Five were excluded from the observation (2 did not give consent, 1 was obese, 1 had severe anemia, and 1 was using midazolam before observation) and one patient was excluded from the analysis for wracking cough during the observation. No hypotension (systolic blood pressure < 60 mmHg), bradycardia (heart rate < 50 bpm), oxygen desaturation (SpO_2_ < 95%), or respiratory depression was observed during the study. There is no significant difference in patient characteristics, liquid volume, or ETCO_2_ between the two groups. Orexin-A levels were significantly higher in the sleep disordered group (Table 1).

**Table 1:** Subject characteristics and concentration of orexin-A

Parameters	Sleep Disordered	Control	*P*
Age (yrs)	50.8 ± 2.9	49.9 ± 4.7	0.708
Body weight (kg)	63.4 ± 6.9	59.8 ± 7.2	0.131
Height (cm)	164 ± 3	161 ± 4	0.055
Glucose (mmol/L)	4.78 ± 0.58	4.96 ± 0.61	0.332
ETCO_2_ (mmHg)	25.4 ± 3.3	26.4 ± 3.2	0.327
Liquid volume (mL)	405 ± 58	393 ± 61	0.536
Orexin-A (pg/mL)	72.2 ± 18.2	36.2 ± 14.2	< 0.001*

Data are presented as means ± SD. ETCO_2_ was recorded before verbal commands. Liquid volume data collection started from the time entering the room until the end of observation. ^*^P < 0.05 versus control

**Fig. 2:**
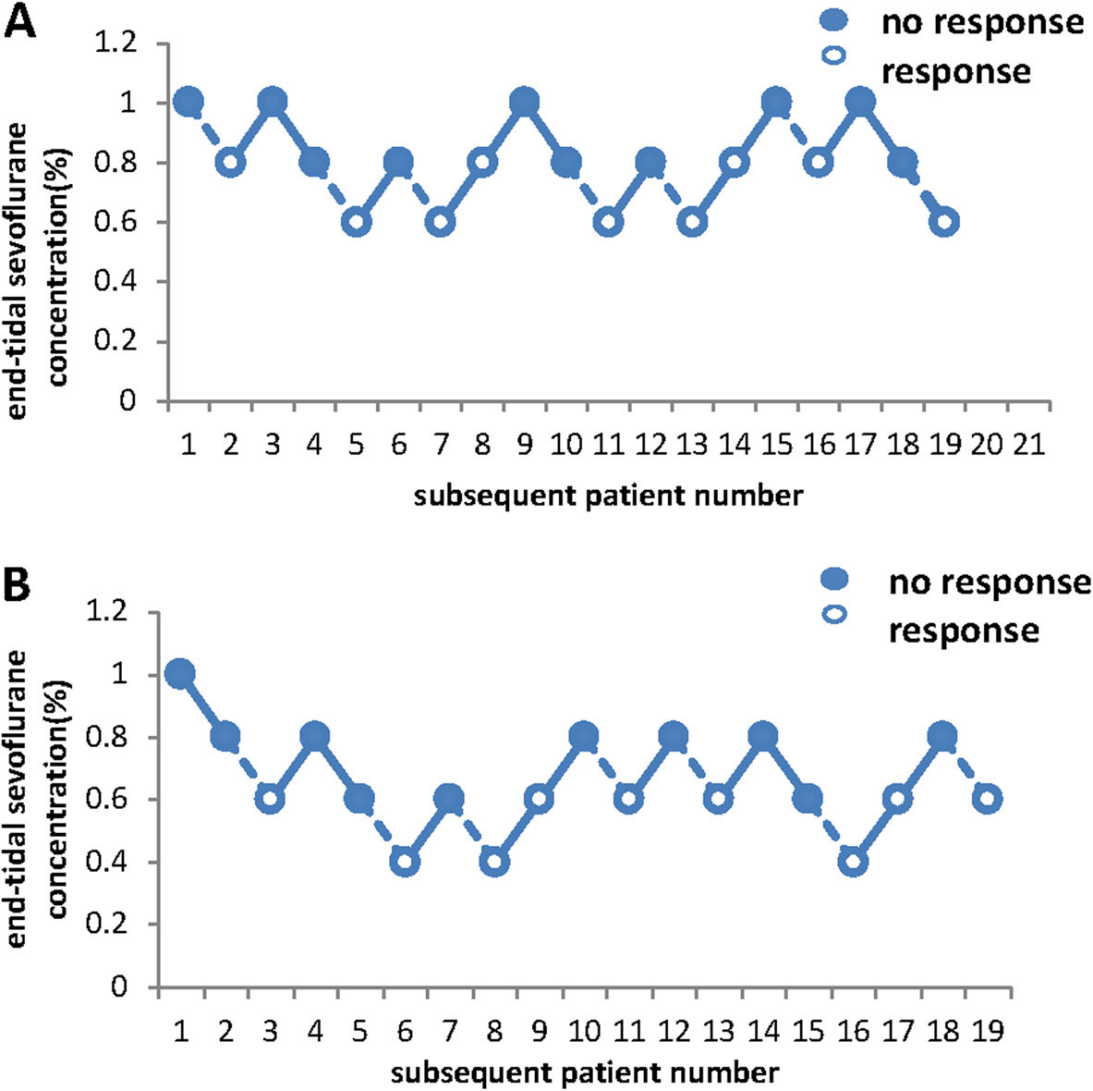
Response of each subject to predetermined end-tidal sevofluane concentrations. Closes circles represent no response to the vocal command and open circles represent response to command. The dotted line between points of and showed seven crossovers of responses. **a**, MACawake of sevoflurane in patients with sleep disorders. **b**, MACawake of sevoflurane in the control group

**Fig. 3:**
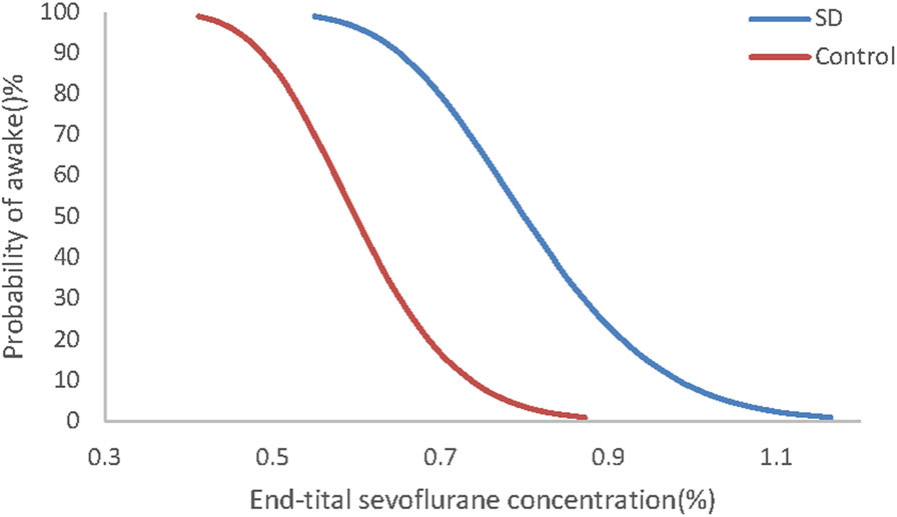
The dose-response curve from the probit analysis of end-tidal sevoflurane concentrations and probability of being awake. Minimum alveolar concentration-awake (MACawake) in patients with sleep disorders was 0.8% [95% CI, 0.683–0.926%]; MACawake in the control group was 0.60% [95% CI, 0.493–0.689%]

Fig. 2 shows the individual responses to the vocal command according to the up and down sequence. The 50% effective dose for MACawake was 0.80% [95% CI, 0.68–0.93%] and 0.60% [95% CI, 0.49–0.69%] for the sleep disordered and control group, respectively, and the relative median potency was 0.749 (95% CI, 0.24–0.97). There was a difference in the median potency of the 2 groups because the CI did not include one. Fig. 3 depicts the dose-response curve of the probability of awake between the two groups. Logistic regression analysis showed that the equation of the logistic regression analysis was Pawakening = 9.595 + 0.078 Orexin-A, with OR of 1.081 (95% CI is 1.020–1.146, *P* = 0.008) (Table 2).

**Table 2:** Results of logistic regression analysis for P_awakening_ as the dependent variable

Independent variable	Regression coefficient (β)	SE	OR	95% CI for OR	*P* value
Constant	9.595	3.684	14,684	–	0.009
Orexin-A	0.078	0.030	1.081	1.020–1.146	0.008
Concentration of sevoflurane (%)	−19.937	6.609	0.000	0.000–0.001	0.003

*CI* confidence interval, *SE* standard error

## Discussion

Our study demonstrated that the MACawake of sevoflurane in 100% oxygen in mild-aged women of breast surgery with sleep disorders was higher compared to those with normal sleep. Additionally, patients with sleep disorders exhibited significantly higher orexin-A levels than normal sleepers. Moreover, a positive correlation between probability of awakening and plasma orexin-A concertation was confirmed.

The Dixon “up and down” method is an effective statistical approach for calculation of MAC [ 12, 13]. Six pairs are optimal for a clinical study, and the reliability increases with an increasing number of pairs [ 14]. Based on the calculated sample size, we stopped the observation after seven pairs of opposite responses in the two groups. We derived the MACawake of patients with normal sleep to be 0.60%, consistent with previous work [15] which demonstrated a MACawake of 0.63%.

As far as we know, there are no quantified studies of MACawake of sevoflurane in humans with sleep disorders. The mechanism of increased awakening concentration of sevoflurane is unclear. There are many factors which can affect the potency of inhaled anesthetics [16], such as age or pathophysiological conditions. However, these have been ruled out in the current study. Finally, we conducted the observation only during anesthesia induction to minimize any surgical confounders. Therefore, the change of hypnotic potency was likely attributed to changes in the functional status of the brain as a consequence of sleep disorders.

The orexin system regulates the sleep–wake cycle [17]. Narcolepsy is caused by impaired orexinergic signaling, as seen by lower levels of orexin-A [18]. Genetic and pharmacological blockades of orexin-mediated signaling impact arousal [6]. Conversely, patients with insomnia disorders exhibit higher orexin-A levels, which is consistent with our study [8]. Previous work has also demonstrated a significant correlation between plasma orexin-A and arousal index or Epworth sleepiness scale score [5, 19]. This relationship highlights the critical role of orexin in maintaining wakefulness. Orexinergic neurons may therefore also affect general anesthesia. Animal studies suggest that orexin signaling can modify the anesthetic state, as activation of the orexinergic signals can significantly promote emergence from inhalation [20] or intravenous [21] anesthesia. Additionally, inhibition of orexinergic signaling prolongs awakening time from anesthesia state, impairs arousal, and increases anesthetic duration [5, 22, 23]. Therefore, we propose that an increase in orexin-A may increase the MACawake in patients with sleep disorders.

This study has several limitations which must be addressed. First, all subjects were middle-aged women, which might lead to Berkson’s bias without an elderly control comparison. Also, elderly patients who are more likely to have sleep disorders and thus have serious complications of general anesthesia should be studied in future investigations. Second, we did not measure orexin-A in the cerebrospinal fluid for ethical reasons. Plasma orexin-A originates from the central nervous system (CNS), the pancreas [24], and the gut [25]. However, release of orexin-A from the hypothalamus would be much greater compared to peripheral tissues [26]. Additionally, orexin-A can rapidly cross the blood–brain barrier [ 27], and thus we were required to assume that plasma levels of orexin-A reflect the CNS release of orexin-A. At last, sleep disorders are distinguished by the PSQI scale, which provide a comprehensive assessment. Thus, the causes and types of sleep disorders were not classified, and we did not distinguish between sleep conditions at home and after admission, but those need to be study in the further research.

## Conclusion

We demonstrate that the MACawake of sevoflurane were significantly increased in mild-aged women of breast surgery with sleep disorders compared to women with normal sleep patterns. The potential mechanism may be related to change of orexin-A levels. Further studies are needed to confirm our results. More researches need to explore that if sleep disorder may change efficacy of other general anesthetics, and whether decrease in hypnotic potency of general anesthetics are associated with postoperative delirium.

## Supplementary information

**Additional file 1.** Experimental data.

## Data Availability

The datasets supporting the conclusions of this article is included within the article (and its Additional file 1).

## Abbreviations

CI: Confidence interval
CNS: Central nervous system
DSM-V: Diagnostic and Statistical Manual of Mental Disorders
ETCO_2_: End-tidal carbon dioxide
HAMA: Hamilton Anxiety Scale
HAMD: Hamilton Depression Scale
MAC: Minimum alveolar concentration
MACawake: MAC of inhaled anesthetic agents to achieve a 50% probability of being awake
PSQI: Pittsburgh Sleep Quality Index
SE: Standard error
